# Cardiac tissue engineering: state-of-the-art methods and outlook

**DOI:** 10.1186/s13036-019-0185-0

**Published:** 2019-06-28

**Authors:** Anh H. Nguyen, Paul Marsh, Lauren Schmiess-Heine, Peter J. Burke, Abraham Lee, Juhyun Lee, Hung Cao

**Affiliations:** 1 Electrical and Computer Engineering Department, University of Alberta, Edmonton, Alberta Canada; 2 Electrical Engineering and Computer Science Department, University of California Irvine, Irvine, CA USA; 3 Biomedical Engineering Department, University of California Irvine, Irvine, CA USA; 4 Chemical Engineering and Materials Science Department, University of California Irvine, Irvine, CA USA; 5 Mechanical and Aerospace Engineering Department, University of California Irvine, Irvine, CA USA; 6 Bioengineering Department, University of Texas at Arlington, Arlington, TX USA; 7 Henry Samueli School of Engineering, University of California, Irvine, USA

**Keywords:** Cardiac tissue engineering, CRISPR/Cas9 systems, 3D scaffolds, Machine learning

## Abstract

The purpose of this review is to assess the state-of-the-art fabrication methods, advances in genome editing, and the use of machine learning to shape the prospective growth in cardiac tissue engineering. Those interdisciplinary emerging innovations would move forward basic research in this field and their clinical applications. The long-entrenched challenges in this field could be addressed by novel 3-dimensional (3D) scaffold substrates for cardiomyocyte (CM) growth and maturation. Stem cell-based therapy through genome editing techniques can repair gene mutation, control better maturation of CMs or even reveal its molecular clock. Finally, machine learning and precision control for improvements of the construct fabrication process and optimization in tissue-specific clonal selections with an outlook of cardiac tissue engineering are also presented.

## Introduction

The adult mammalian heart is among the least regenerative organs thus cardiomyocytes (CMs) are threatened by a multitude of factors; such as necrosis, apoptosis, and oncosis (or ischemic cell death), which may lead to heart failure [1, 2]. Necrosis, or premature cell death due to physical or chemical injury, and apoptosis, or programmed cell death, have more recently been found to be linked together during pathological states of heart disease [3]. Regarding cardiac pathogenesis, myocardial infarction results in scar tissue, regions where CMs are replaced with fibrillar collagen and/or fibroblast-like cells [4]. Oncosis, or ischemic cell death, is recognized as distinct from necrosis in that the cell swells instead of shrinks, but necrosis and oncosis both follow cell injury [5]. Heart failure, as of 2017, affected about 38 million people globally [6], and 6.5 million of those are in the U.S. alone [7]. Besides heart pathogenesis, the risk of heart disease rises steadily and sharply with age [8]. All of these factors compete with the low cell turnover rates of mature mammalian CMs, which is somewhere around 0.3–1% annually [6]. For these reasons and more, the heart is one of the most important topics for tissue engineering research. These researches not only would reveal mechanism of cardiac repair and improvement of cardiac function through tissue engineering that provide new scientific insights, but also propel forward the findings to new therapeutic designs for clinical treatment.

To date, although cardiac tissue engineering has not been absolutely ready for routine clinical applications, autologous and allogeneic adult stem cell transplants have been successfully in cardiac therapies with randomized clinical trials (RCTs) in some reported cases [9]. Therefore, engineering innovations hold promise to shape research and treatment directions in the years to come. Together with tissue-engineered hearts for transplantation, current methods have been focused on stem cell transplantation in which cells are seeded onto 3D polymer scaffolds followed by electrical, mechanical or chemical stimulation (heparin and hyaluronic acid) in order to promote stem cell differentiation. Eventually, the diseased and injured heart tissues are expected to restore [10–12]. However, the concerns of histocompatibility of regenerated cardiac cells and stem cell-derived pro-arrhythmic substrates [13, 14] have limited the use of stem cell-based therapies for human heart failure. As a result, immune tolerance and growth of stem cells on novel biomaterials have recently emerged as a promising approach for cardiac repair [12]. Interestingly, recent findings in molecular mechanisms during the developmental stages of mammalian hearts have suggested that new CMs may arise from existing CMs and progenitor or stem cells at early stages of embryo and newborn development [15–19]. Toward this end, stem cells, including cardiac stem cells (CSCs) [20], embryonic stem cells [21], bone marrow-derived mesenchymal stem cells [22], and cord-derived mesenchymal stem cells [23] are essential materials for cell-based tissue engineering applications; which have already entered the clinical setting with some challenges [24–26]. However, the capacity and significance of adult mammalian cardiomyocytes and CSCs regeneration remain controversial [27–30]. One of reasons is that specific stem cell markers that are used to identified CSCs, such as c-KIT, are necessary but not sufficient for their identification [31–33]. Recently, Kretzschmar et al., have used single-cell mRNA sequencing and genetic lineage tracing to interrogate existence of CSCs with unbiased mouse models of proliferation and they found that cycling cardiomyocytes only dominantly presented in the early postnatal growth phase [27, 32], while many noncardiac cell types mainly present in damaged adult myocardium [27, 34]. Although the gene expression profile was shown the same in both injury-activated cardiac fibroblasts and neonatal cardiac fibroblasts under in an autocrine fashion, there is no evidence of a latent CSC population [32]. Although the presence of CSC population in adult hearts is still controversial, differentiating other stem cells into mature cardiomyocytes is attractive in cardiac therapies.

To get a high yield of mature cardiomyocytes, scaffolding and its derivates of growth factor/stimulating devices have been deployed as a support substrate for cell growth and transplantation to the host tissue in regenerative medicine [35, 36]. For instance, cell alignment is essential for cardiovascular tissues in order to maintain the microarchitecture and biological functions; therefore, various strategies have been developed to induce cardiac cell alignment. Those methods include topographical patterning (e.g., micro- and nano-grooves and aligned nanofibers), chemical treatment (patterns with cell-adhesive or repellent chemistries), controlled stress/strain conditions (e.g., stretching, fluid shear stress, and compression), and a combination of them [13, 14]. In its early stage, tissue engineering research involving CMs revolved around injection of differentiated stem cells with the hope they would grow and synchronize with the host [6]. However, it was found that these cells required environmental conditions which were biomimetic to early cell growth conditions, in order to differentiate and bind into a syncytium [15]. This could be pulsatile electrical stimulation similar to native syncytium electric fields [15], simultaneous electrical stimulation and cyclic mechanical stretching [37], or any combination of these with bioinspired antioxidant materials and other microenvironment cues [12, 17], which can be optimized by algorithms based on experimental datasets.

The recent rise of artificial intelligence, especially machine learning and deep learning, has paved the way for a wide range of applications, and cardiac tissue engineering is not an exception. Machine learning (ML) aims to develop algorithms that discover trends and patterns in existing data and use this information to make predictions on new data. ML has proven to be of great potential value in a variety of application domains, including biological investigations and healthcare where accurate analysis of biomedical data benefits early prediction and detection of diseases [38]. ML encompasses a diverse set of schemes by which a machine extracts certain features, “learns” the pattern of features associated with a certain group and then predicts the group based on feature patterns of new samples. The ML methods are particularly effective in situations where prediction involves large data sets, especially datasets of terabyte or petabyte size [39]. Specifically, ML algorithms can perform efficient data training to identify relationships of inputs and outputs, although there are not typically intuitive interpretations for how hidden layers in these algorithms operate [40]. However, in this field, it is still in the proof-of-concept phase where structures and algorithms have been focused in order to minimize or eliminate human intervention in these processes. For example, ML has been used for automated drug classification based on contractility of human pluripotent stem cell-derived engineered cardiac tissue [41], protein-ligand binding affinity [42], and histopathological image analysis [43]. Regarding 3D scaffold constructs, the fabrication could be controlled and optimized with an adaptive neuro fuzzy inference system and a Pareto-based self-learning evolutionary algorithm [44].

In addition to many strategies for precision control of myocardial microenvironment of smart biomaterial scaffold for cellular adhesion, growth, and maturation [45, 46], ML and evolutionary algorithms have been used to identify stemness features associated with oncogenic dedifferentiation [47], 3D scaffold design [48], local microenvironment changes, and to drive cellular differentiation pathways in CM maturation. Artificial intelligence-based approaches, such as machine learning and deep learning, refer to a set computer programs that deal with data training and perform intelligent analysis [49–51]. Machine learning is an integration of algorithms such as naïve Bayesian [52], support vector machines (SVM) and updating deep neural networks which are highly dependent on high-quality data. ML with the model of end-to-end (E2E) increases levels of accuracy of the process from big datasets created from high-throughput screening data for drug discovery and development [53]. Recently, deep learning as part of machine learning methods has catalyzed interest for drug discovery [54]. Deep neural networks approaches [55, 56] can process with all combinatorial variations using the single E2E black-box network or the deep classification network [57], which were deployed for biomedical researches in cardiac contractile dysfunction and arrhythmia [58, 59], facial phenotypes of genetic disorders [60], precision phenotyping and clinical diagnostic support systems [53]. In tissue engineering field, it was reported that smart scaffolds integrated with a wireless ML-driven sensing responded to changes of electrophysiological phenotypes, local tissue microenvironment (e.g. pH, protease activity, and biosignatures) [61], and CM phenotyping (e.g. β-Adrenergic receptor) [62, 63]. This may allow training the data for self-repair approaches in the design of 3D scaffolds and cardiac regeneration. Moreover, ML allows performing multifunction by controlling serial signals of the biomimetic paracrine in custom design to identify cell shape phenotypes associated with microenvironment cues [64, 65]. Thus, novel ML-based scaffold designs may provide not only a robust substrate for cardiac tissue culture but also a real-time database for precision bioactive control (e.g., timed release of growth factors) in the microenvironment that may be required for improvements of CM regeneration and repair.

In the next sections of this paper, molecular and biomaterial engineering approaches will be introduced and discussed followed by methods for nano-scaffold fabrication. Updates of upcoming and ongoing ML applications in tissue engineering, especially as it relates to cardiac tissue engineering, will be then broadly covered.

## Genome editing and stem cell differentiation

### CRISPR/Cas systems for cardiac tissue engineering

#### Gene mutants in human cardiac failure

According to statistics, it was revealed that gene-related factors and genetic variations are responsible for complex forms of cardiovascular disease (CVD) [7]. For example, genetic variants of missense mutations (T983I) in the KCNH2 (LQT2) gene frequently relate to and arrhythmogenic disorders like QT syndrome [18]. Techniques using induced pluripotent stem cells (iPSCs) and genome editing can intervene at molecular levels for cell adhesion, differentiation, and cell alignment in cardiac tissue engineering [19, 66]. Genome editing based on programmable nucleases is a molecular process that uses clustered regularly interspaced short palindromic repeats systems (CRISPR) with Caspase 9 (Cas9) guiding enzymes and has been used to introduce the catecholaminergic polymorphic ventricular tachycardia type 1 (CPVT1) associated cardiac ryanodine receptor 2(RYR2) mutation in healthy wild iPSCs [19]. In principle, CRISPR/Cas9 systems are nucleic acid-targeting defensive tools of prokaryotes, whose operation is exploited to edit mammalian genomic materials and control transcriptional regulation of endogenous genes; in turn, these genes can be used to control molecular routines in tissue regeneration [67]. By introducing F2483I RYR2 mutations to wild type human iPSCs (hiPSCs), calcium signaling pathology can be observed and compared between iPSC-derived CMs from CPVT1 patient cells and gene-edited cells. Results show that increased diastolic Ca^2+^ and reduced sarcoplasmic reticulum store size in gene-edited and patient-derived CMs are consistent with each other [19]. Alternatively, CRISPR/Cas9 engineered R453C-βMHC [68] and corrected PRKAG2 mutations in patients [69] allow them to recover physiological mitochondrial functions, as well as electrophysiological and structural abnormalities, making this a reasonable approach to recover CM functionality [68, 69].

#### Potential of CRISPR/Cas systems in cardiac tissue engineering

The CRISPR/Cas9 system is based on two components: a synthetic, single-stranded guide RNA (sgRNA) and Cas9 enzymes. The spacer part of the sgRNA can be designed to bind complementary DNA targets for Cas9 cleavage at a protospacer adjacent motif (PAM) in the DNA targets, in order to generate a single-strand or double-strand break. Subsequently, a new DNA is formed through one of the two molecular mechanisms: non-homologous end joining (NHEJ) or homology directed repair (HDR). These mechanisms serve to introduce random mutations and to precisely edit DNA sequences, respectively [70]. However, several challenges exist with the use of this system, such as off-target effects and the difficulty in delivery of large Cas9 sequences. Off-target effects refer to nonspecific and mismatched genetic modifications that can arise using engineered programmable nuclease techniques. In CRISPR/Cas9 systems, these off-target effects can be resolved by reducing non-specific binding of gRNA sequences. CRISPR/Cas9 systems can be introduced to cells in the form of plasmid DNA, RNA, or proteins, which can be used for engineering cells in cardiac tissue regeneration [68, 71]. Recently, Doudna et al. explored CasX enzymes risen from a TnpB-type transposase, a distinct family of RNA-guided genome editor (CRISPR/CasX), that can be used as a third platform for RNA-programmed genome editing [72]. With the compact size, dominant RNA content, and minimal trans-cleavage activity, CasX is the smaller size compared to that of the previous reported Cas9 and Cas12a. This provides an increased efficiency of therapeutic delivery and overcoming the human immune systems, which may offer more advantages relative to current CRISPR/Cas systems. CRISPR/Cas systems can be also utilized to reactivate non-dividing cells and terminally differentiated mammalian cells, or change cell structures on-demand to address tissue architecture formation, both of which having been demonstrated for cardiac stem cell engineering [67–69]. Moreover, due to difficulty in ex vivo culture of primary CMs, a potential alternative approach is using a CRISPR/Cas9 system to edit iPSCs-derived CMs in situ. These edited iPSCs can differentiate into readily transplantable cells: iPSC-cardiac progenitors or iPSC- derived CMs to deliver to the diseased heart though intracoronary or intramyocardial routes. As an example, iPSC-derived CMs have been seeded on micro-threads then transferred to cardiac tissue and contractile cardiac fibers [73]. Unfortunately, iPSC-derived CMs are immature with regards to their structure and function, and this immaturity has narrowed down their applications in drug screening and cell-based therapies [74]. One of solutions is to create the geometry of the environment based on extracellular matrix (ECM) for cellular behavior and maturation [75].

Attachment of CMs or iPSC-cardiac progenitors to culture systems is highly dependent on levels of fibronectin and collagen IV in the extracellular matrix (ECM), both of which feature prominently in cardiac cell fate [61]. With the CRISPR/Cas9 system, the expression of those matrix proteins can be increased, which improves cell homing functions in culture systems. In another report, this editing tool has been used to eliminate inactivated genes in mature CMs through the Adeno-associated virus 9 (AAV9)- sgRNAs system [76]; it has also been used for editing the mitochondrial genome in order to control membrane potential disruption and cell growth inhibition, which are related to cancer genesis in transplanted tissues [40]. Moreover, the CRISPR/Cas9 system has been applied to human stem cell-derived CMs for cardiovascular disease modeling and cardiotoxicity screening; enabling studies of new cardiovascular disease treatments and drug-induced cardiotoxicity [77]. In addition, the CRISPR/Cas9 system can address safety concerns by reducing immunogenicity and even the risk of arrhythmia by removing the mutant ryanodine receptor 2 (RYP2) from the multimeric complexes [78]. To minimize the risk of immunogenicity, in addition, the suicidal thymidine kinase gene can be induced into the genome of stem cells for iPSCs and embryonic stem cells (ESCs) to efficiently protect hESC-derived allografts from immune rejection [66, 79]. Molecular activities of ion channels and gap junctions determine the functionally proficient electromechanical coupling between myocardial cells. Defects in the molecular activities responsible for restoring myocardial electrical conduction can be mitigated by targeted genes [80] and macrophage cell therapy [81]. Macrophages are innate immune cells that reside and accumulate in the healthy and injured hearts. A complex crosstalk between cardiomyocytes and macrophages regulates the fate of cardiomyocytes in the injured heart and plays central roles in cardiac hypertrophy [82].

Given that the clear majority of heterogeneous CMs in postnatal tissue is postmitotic, a new routine for homologous recombination of these cells is required. This begins by analyzing the transcriptome during the differentiation process of human PCSs to mature CMs in order to identify a key transcriptional roadmap for molecular intervention [35]. Interestingly, CRISPR/Cas9 systems can contribute to cell differentiations by controlling the gene profile expression through Cas activity. Polstein et al. reported a light-inducible CRISPR/Cas9 system to control endogenous gene activation and transcription [83, 84]. Alternatively, CRISPR/Cas9 systems provide direct benefits in controlling of immune response for CM engraftment [85]. Since mature CMs are postmitotic cells, they lack the HDR repairing mechanism and the CRISPR/Cas9 system doesn’t work in these cells. This restriction can be overcome with iPSC-CMs from patients or endothelial cells (ECs), smooth muscle, and cardiac progenitor cells in which genes of interest are edited ex vivo. Then these cells can differentiate to all cardiac lineages used for cardiac regeneration. In addition, together with synthetic biology, bioinformatics, and deep learning CRISPR/Cas9 systems are able to reduce off-target consequences and create gene regulatory networks for multicellular development [61, 86]. Using CRISPR/Cas9 systems to reprogram fibroblasts into skeletal myocytes with the targeted activation of the endogenous Myod1 gene locus results in elevated expression levels of myogenic markers, mainly because activation is comparable to a lentiviral vector-delivered MYOD1 transcription factor [87]. With such an activation, in vivo CMs and other cardiac lineages at injury sites can be converted from cardiac resident fibroblasts. This process relates to the complex multilayered regulatory systems that induce cell differentiation and heart development as a system biology level [88].

**Fig. 1:**
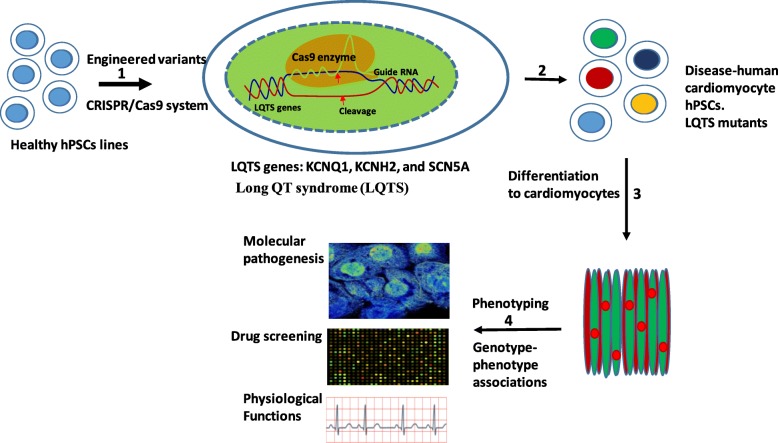
(1) Introduction of LQTS genes in independent healthy hPSC lines using CRISPR/Cas9. (2) Generation of disease-cardiomyocyte hiPSCs. (3) Isogenic sets of hPSC-CMs were differentiated from the edited hiPSCs lines. (4) Molecular analysis and phenotyping of hPSC-CMs (upper) molecular pathogenesis, (middle) drug screening, and (bottom) physiological functions

Gene regulatory networks play an important role in the spatiotemporal expression of desired cardiac regeneration-related proteins. Products of this expression are involved in many endogenous and exogenous physio-chemical stimuli, producing growth factors and other cytokines which shape cardiac tissue structure. The GRN can be regulated at molecular levels via the technique of synthetic biology coupled with bioinformatics, in order to design biological circuits and provide tools for more intricate control of cellular functions. With such an approach, tissue regeneration can overcome long-standing challenges and introduce new methods for basic research and clinical applications. In biosafety regulations, CRISPR/Cas9 system activity could be eliminated to avoid risks of permanent expression of foreign targets when designing tissue structures for clinical use. Figure 1 introduces a protocol to edit mutant genes in hiPSCs and monitor cardiac differentiation; which was done with molecular and phenotypic characteristic measurement. Briefly, CRISPR/Cas9 system was used to introduce long-QT syndrome genes in independent healthy hiPSC lines to generate disease-CM hiPSCs. This resulted in the formation of isogenic sets of hiPSC-CM which were characterized with phenotyping and molecular analysis. CRISPR/Cas9 systems for tissue-specific engineering of stem cells not only provide new avenues for functional tissue engineering and regenerative medicine, but also control the immunological balance in both the early and chronic stages after cardiac injury [89]. Proinflammatory cytokines present in increased levels in diseased and injured tissues, which leads to the increase of tissue degradation and can prevent differentiation of hiPSCs [90]. Recently, reports strongly suggested that controlling inflammatory cytokine secretion from resident cardiomyocytes and cell interaction is one potential approach for cardiac angiogenesis and cellular regeneration [91, 92].

Previous studies have reported that transplantation of cells genetically engineered for constitutive overexpression of interleukin 1 receptor antagonist (IL-1Ra) is effective when creating cells-integrated scaffolds for implantation [93]. This approach also provides great promise in combating inflammatory levels of interleukin 1 (IL-1), a challenge for transplanted and/or engineered tissues. To this end, RNA interference or CRISPR/Cas9 systems have been used for controlling the expression of inflammatory cytokines [43]. Alternatively, regulation of gene expression of growth factors and anti-inflammatory cytokines (IL-4, IL-1Ra, and IL-10) in cell-based engineering platforms are also a considerable approach. Compared to RNAi technology, however, the CRISPR/Cas9 systems provide permanent removal of inflammatory cytokines from the cell genome, this guarantee long term control of anti-inflammation in cardiac tissue regeneration.

Due to numerous challenges in current cardiac tissue regeneration, the CRISPR/Cas9 system has become an effective alternative which can tackle those by providing complex genome editing and transcription regulation, in order to control differentiation, at genomic and molecular levels [67, 70]. While still in its early stages, ongoing research on the use of CRISPR/Cas9 systems for more-complex implementation of the CM molecular clock [94] by controlling the transcription-translation feedback loop may be a milestone in tissue engineering. In brief, CRISPR/Cas9 systems hold potentials to dramatically improve comprehension of cellular processes and contribute significantly to cardiac tissue engineering.

### Stem cell differentiation

Differentiation of stem-cell-derived CMs into the desired lineages requires many aspects of the scaffold constructs, cell fate, and cell’s environment [36, 73, 95–98]. Using hiPSCs to differentiate into mature CMs has been considered as a potential approach towards therapeutics in cardiac tissue generation. With optimal protocols, fetal hiPSCs can be differentiated into almost 100% pure CMs. Although human ESC-derived CMs are a predominant source of adult human cardiac myocyte for clinical therapeutics, they still lack many essential features such as being well-organized and distributed, and functional transverse tubules (T-tubules) [99]. Chong et al. reported that mature human ESC-derived CMs, rather than immature, may become the preferred candidate to reduce the risk of arrhythmias in the transplantation therapy [100]. In addition, adult-like hiPSC- derived CMs can be widely used for applications in stem cell-based disease modeling and in drug toxicity screening [95, 101]. Some strategies of generating cardiac tissue from stem cell-derived CMs, in which their cellular morphology is similar to human adult cardiac structure and function, have been reported [74, 102, 103]. Ronaldson-Bouchard et al. used different stages (day 12 and day 24 differentiation) of hiPSC-derived CMs and co-cultured them with fibroblasts in a fibrin-based hydrogel to grow mature cardiac tissues around two flexible pillars [104]. These pillars were used to induce forces in the contracting tissues, as forces are observed in native myocardium. After 1 week in culture, either constant electrical stimulation (2 Hz for 3 weeks) or intensity training (2 to 6 Hz ramp over 2 weeks, then back to 2 Hz for one week) were applied to stimulate the differentiation and growth of hiPSCs to maturize CMs, which were determined through the molecular, cellular, and functional level of the differentiation [104, 105]. At the molecular level, genes associated with adult-like conduction, atrial isoform-related ventricular isoform of myosin, ATP production, and calcium transportation were highly expressed, which indicated maturation. At the cellular level, growth of CMs with ordered sarcomeres and a high density of mitochondria, were observed [104]. Vital proteins such as T-tubules and folding of the sarcolemma membrane, involved in calcium transportations, were found in the cell [106, 107]. Cell alignment in tissue constructs, where cells were adhered to one another with mechanical strength at gap junctions, promoted electrical signaling transmission between cells in the constructs. Well-aligned hiPSC-derived ventricular CMs on the human ventricular cardiac anisotropic sheet, a cardiomimetic biohybrid material, was reported in fully key electrophysiological features of native human ventricle [108]. This was observed only when hiPSC-CMs received an intensity training at an early stage [109]. After spending the intensity training, cardiac tissues were able to efficiently perform action potentials through a process of excitation-contraction coupling. Electrical stimulation (excitation) induces mechanical response (contraction), which allows myocardium to contract. Wiegerinck et al. reported that increased beating frequency was the simultaneous result of increased contraction force and faster relaxation [110]. Various regulatory factors involved in CM maturation, hormone-driven cues [99], intensive electrical stimulation [111, 112], cell composition and matrix/media [113, 114] have shown the most potential to achieve hiPSC-derived CMs in scaffold environments.

In cardiac tissue engineering, natural polymer scaffolds play an important role in promoting differentiation and growth of hiPSC-derived CMs owing to their minimal immunogenicity and biodegradability. Kaiser et al. used a blended fibrin and collagen scaffold to differentiate hiPSC-derived CMs into engineered myocardium [97]. Results showed that expression of cardiac troponin T (cTnT) in CM populations were dependent on the scaffold compaction. While the decreased compaction showed the lowest (24.4%) and highest (60.2%) positive expression of cTnT^+^ CM purities, the highest compaction showed 40–50% cTnT^+^ population [97]. This study clarifies the correlation of hiPSC-derived CMs and scaffold interactions and provides a basis for integrated design of customized scaffold constructs for cardiac tissue engineering.

## Biomaterials and 3D scaffold fabrication

### Characteristics of biomaterials

Biomaterials in the forms of hydrogels, carriers, and scaffolds play a vital role in anchoring cells and helping them generate into functional tissues [115–117]. Although those forms have different specific patterns in tissue engineering, all of them serve as a framework substance for proliferation and differentiation of the desired tissue. For example, carrier materials enable cells or chondrons to produce the ECM that holds growth factors in skin wound healing and cardiac remodeling and repair [118, 119]. Porous hydrogels entrap embedded cells and allow diffusion of gas and metabolites through their pore network [120, 121]. Similarly, scaffolds are also porous matrices, though they allow cell migration and attachment to the damaged tissue, as well as act as a substitute for lost tissue in the body [122]. The developing highly-porous scaffold biomaterials significantly depend on their types of materials, functionalization, and geometry.

Typically, biomaterials for tissue engineering are synthesized or modified from primary natural materials, then further processes are conducted to form appropriate morphology and characteristics for a desired application. They include polyglycolic acid (PGA) [123], poly(L)-lactic acid (PLA), poly(DL) glycolate (PLGA), and polyvinyl alcohol and their derivatives [124–126]. In contrast, natural biomaterials include collagens, alginate, chitosan, fibrin and hyaluronic acids. Recently, advances in synthetic chemistry have contributed to novel hybrid biomaterials with outstanding properties in terms of conductivity and strength [127, 128]. For use in cardiac tissue engineering, it is required for biomaterials to support tissue reconstruction and regeneration via active support for cell-to-tissue processes by promoting cell-cell adhesion, proliferation and differentiation. These biomaterials can also culture healthy tissues by forming three-dimensional structures for gas and nutrient transportation as well as formation of vascular supportive substructures for blood vessels. The biomaterials used for scaffold fabrication processes can optimize constructs used in clinical settings; allowing for maximizing cellular adhesion space, ECM secretion, revascularization, and paracrine processes.

### Shaping biomaterials in 3D structures

Scaffold materials play a key role in tissue engineering and have been used more and more in clinical practice [129–131]. These materials form a biomimetic ECM which promotes cell adhesion and differentiation, as well as 3D organotypic cultures [132]. By combining modern advances of three major fabrication techniques, namely electrospinning, self-assembled monolayers, and thermally induced phase separation, with peptides and DNA, biomimetic 3D scaffolds have been developed for CM regeneration [133–135]. These systems support differentiation of various stem cells down multiple lineages and create relevant 3D specific tissues for clinical practice.

Obviously, specific cell types could be seeded on the biomimetic nanofibrous scaffold to regenerate desired tissues. Both primary and stem cells can be used, for different purposes [36, 98, 112]. Primary cells are collected directly from mature tissue and cultured to obtain the desired cell number and form tissue constructs. However, quick phenotypic changes, limited proliferation numbers, and aging of primary cells inhibit their use once the cells are transferred from their natural living conditions to artificial ones [132, 136]. While CMs can be taken from specific tissue sources for targeted applications, robust scaffolds and engineered biological tissues are needed to improve to CM characteristics in new implanting environments. Most scaffolds used for cardiac tissue engineering are hydrogel materials and 3D nanofiber matrices, which feature benefits such as controlled release of growth factors and good electrical conductivity [137, 138]. Results from confocal laser scanning microscopy, scanning probe nano-tomography, and transmission electron microscopy show that cardiac cells and fibroblasts actively interact with 3D nanofibrous substrates, but in different ways [139]. While fibroblasts make contact with nanofibers through focal adhesion clusters, without wrapping the fiber, CMs develop a distinguished sheath structure and covering fiber to increase contact area [139, 140]. These results point to a new perspective on how cultured cells interact with 3D nanofibrous scaffolds. A host of previous studies reported that matrix anisotropy and stiffness predominantly influence 3D structural cell phenotypes, cell migration, proliferation, and differentiation of cultured CMs [141]. Cardiac cells grown in 3D matrices were always in tight contact with each other through cellular junctions, which results in considerable mechanical adhesion between cardiac cells and fibers. The increase in mechanical adhesion was found to be linked with the increased contact area between the cells and fibrous structures [142]. The contact area plays a role for focal adhesion kinase in cardiac mitochondrial biogenesis induced by mechanical stress, which contributes to the hypertrophic growth of cardiomyocytes via control of mitochondrial transcription cascade [143].

Cellular parameters like the number of mitochondria and endoplasmic reticulum membranes featured higher counts of cells grown in 2D constructs. Moreover, Wobma and colleagues reported that upgraded “smart” scaffolds can directly control biologically active molecules like hormones in the paracrine pathways directly through the cell membrane, avoiding dissipation through the whole tissue solution [144]. In such a system, bioactive molecules are efficiently used for CMs because they increase the diffusion of these molecules from neighboring cells through paracrine hormones. It is also helpful if conducting materials are integrated into these platforms prior to cardiac cell regeneration. Fibers are immersed in cardiac cells to promote high densities of electrical contacts, thus forming an electrical network on the outer part of the nanofibrous structures isolated from the surrounding integrin microdomains. With currently-available biomimetic models [129], the physical basis for this could be explained with van der Waals forces and DLVO theory. DLVO theory is the typical explanation of the stability of colloids in suspension [145]. The explanation of the cell interaction stability is governed by physical and chemical interactions between cellular surfaces that the balance between two opposing forces-electrostatic repulsion and van der Waals attraction is under DLVO theory [146, 147]. The interaction energy is calculated by the sum of van der Waal forces and electric repulsion energy; thus zeta potential, hydrodynamic diameter, and cellular surface thermodynamic properties play an important role in the interaction energy in the scaffold microenvironment for cell alignment and elongation [148].

Model of generation, alignment, and stabilization of spindle shaped fibroblasts and vessel under oscillatory stretch was also reported [149]. These results reveal a new mechanism for vessel network formation: under oscillatory strain, 3D scaffolds can promote mural cell alignment, cell proliferation, translocation of a mechanosensitive transcriptional activator (YAP) into cell nuclei, and increased expression levels of β-catenin. This directs ECM alignment along the orientation of the fibroblasts. Furthermore, ECs, which are tolerant to stretch stimulus, form aligned vessels directed by the fibroblast and ECM alignment. However, there is loss of fibroblast alignment and vessel alignment due to mechanical uncoupling of the cells after adding blebbistatin to the culture medium [149]. In addition, both fibroblasts and vessels lose alignment when the cellular proliferation and signaling pathways responding to mechanical stimulus are inhibited. Stretch stimulus promotes the stable production of growth factors, which enhances mural cell differentiation, thereby enriching stability and alignment. These findings demonstrate how increased mechanical strain affects cell development, differentiation, and shape formation during the vascularization process. Cellular stretching is restricted by nucleus size, which is less sensitive to deformation [139]. At the adhesive site, the cell is stretched by surface tension force. Absorbing fibers is not energetically beneficial in the case of the actin cytoskeleton, hence contact is minimized with fibers by reduction of cell membrane surface area [150]. Thus, these cells are able to generate enough forces to overcome the resistance of the actin cortex at several filament assembly complex locations. In contrast to fibroblasts, CMs contain integrins in costamere structures that anchor sarcomeres to the ECM, so myocytes have much higher affinity with the substrate and serve to stabilize areas of cell-ECM interaction. Therefore, when CMs grow on suspended fibers, the myofibrils start attaching and aligning with them to increase the area of interaction with the substrate [139].

The 3D microenvironment increases adherence and direct reprogramming of fibroblasts into CMs throughout the matrix via a metalloproteinase dependent mechanism [151]. The nanofibrous poly(L-lactide) (PLLA) scaffolds adsorb serum proteins and ECM proteins like fibronectin, vitronectin, and laminin at quantities four times higher than solid walled PLLA scaffolds [151, 152]. In nanofibrous form, the absorption of protein is influenced by many surface characteristics such as protein absorption layers, surface-to-volume ratio, surface nm-scale morphology, crystallinity, and orientation of the polymer in its nanofibrous form. Finally, nanofibrous scaffolds promote cell adhesion in many cell types, giving them an advantage over solid walled scaffolds.

### 3D-gel of hybrid biomaterials

**Fig. 2:**
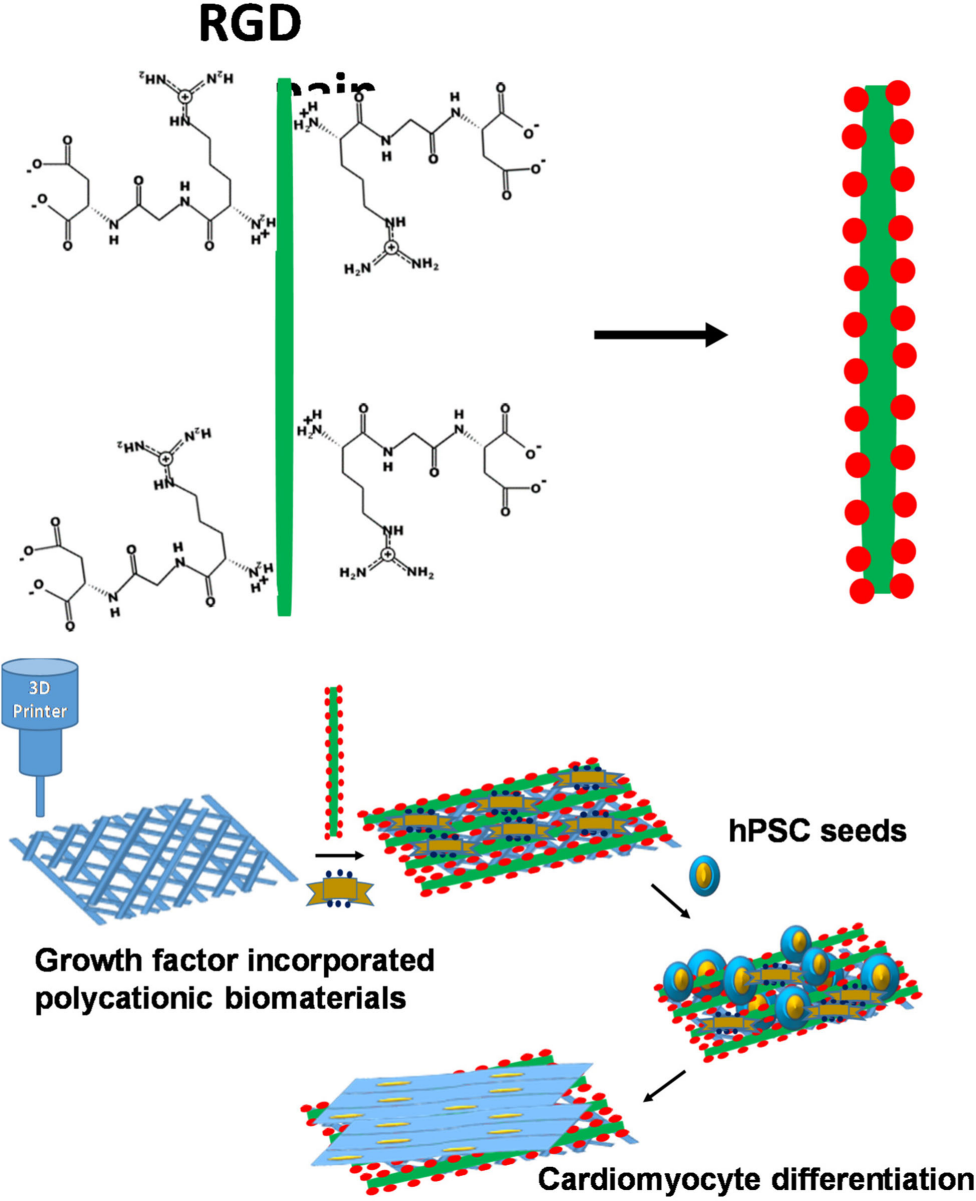
Biomaterials are based on self-assembled monolayers from bacteriophage display for 3D scaffolds formation. (Top), RGD peptide is displayed and fused to the solvent-exposed terminal of each copy of major coat protein (pVIII) through genetic engineering. The side wall of filamentous phage by RGD-coding gene into gene VIII to generate RGD-phage. (Bottom) The 3D scaffold of RGD-phage nanofibers (negatively charged) self-assemblies with polycationic biomaterials and integrated into a 3D printed bio-ceramic scaffold [156], which electrically stabilizes the phage nanofiber inside the scaffold. The resulted scaffold is seeded with hiPSCs and the implanted into cardiac defect. The presence of RGD-phage in the scaffold induced the formation of cardiomyocytes [157]

Natural biomaterials can be produced from self-assembled monolayers (SAM) of different polymers through hydrogen bonds, van der Waals forces, and hydrophobic and electrostatic interactions [153]. SAM fabrication is very useful and robust, thus some recent studies have attempted to mimic collagen structures from ECM-derived binding peptides, which increased cell adhesion and cardiac repair by cardiac progenitor cells [154]. These systems can work with other self-assembling materials like phage display peptides and genetic materials to improve adhesion, proliferation, and controlled differentiation; rendering many applications in tissue engineering [155]. Wang et al. reported a procedure to fabricate biomaterials for 3D scaffold formation based on SAMs from bacteriophage display [156]. In this approach, a panel of desired peptides was displayed on M13 phages, a bacteriophage of *Escherichia coli*, for the purpose of CM generation by activating ligand-linked microenvironments in damaged cardiac tissues (Fig. 2) [150]. As seen in Fig. 2, RGD and DLEFIFEER ligand motifs that mediate adhesion to the cell adhesive receptors were displayed on major coat protein pVIII and determined through an interaction between nephronectin and α8β1 integrin receptor [158]. Using a 3D printer, assembly of the short peptide-coated nanoparticles into a 3D functional structure was driven by noncovalent interactions to form a scaffold [158]. The mechanisms of these self-assembled processes have led to major advances in the understanding of biological and chemical 3D folding processes for biomimetic supramolecular peptide assemblies in coatings, gels and electroactive materials. The specific function of these materials relies on their helical peptides, β strand peptides, and surface binding monolayer-forming peptides, which electrically stabilized the phage nanofiber inside the RGD-phage scaffold. Subsequently, hiPSCs were seeded in the RGD-phage scaffold and induced the formation of cardiomyocytes [159].

The geometry of the scaffold substrate is very important in cardiovascular tissue engineering because the cardiac tissues need to be highly differentiated to perform high specific functionality. For example, the microscopic level of heart valve needs to be at anisotropic geometry, in order to have particular shape of semilunar valves at the macroscopic level [160]. Microenvironment and contraction properties of cardiomyocytes can be influenced by morphology and mechanical properties by increasing the modulus in the range of 1–30 kPa of 2D substrates [161]. Developing these properties in synthetic 3D scaffold may provide a significant means of controlling cell fate both in vitro and in vivo. An ideal polyester biomaterial elastomer for cardiac tissue engineering should exhibits a relatively-low Young’s modulus, with high elongation and tensile strength [162]. Through a one-step polycondensation reaction and ultraviolet reaction, poly(octamethylene maleate (anhydride) 1,2,4-butanetricarboxylate) (124 polymer) is formed the prepolymer gel and a cross-linked elastomer with highly elastic and tunable properties [162], of which they are dependent on the UV light exposure, monomer composition, and porosity of the cured elastomer. Interestingly, the material does not only provide its elastomeric properties falling within the range of those of adult heart myocardium, but also is optimized for higher elasticity for cardiac cell attachment and interaction in vitro and in vivo [162]. Finally, the polymer expressed relatively-stable degradation characteristics that support potential tissue implants. Recently, Shiekh et al. developed and evaluated an elastomeric antioxidant polyurethane (PUAO) for cardiomyocyte functionality [12]. A serial analysis including uniaxial and cyclic tensile testing, thermal analysis, cytotoxicity, antioxidant analysis, and degradation reveals that PUAO reduces intracellular oxidative stress in H9C2 cardiomyocytes and neutralized reactive oxygen species (ROS) promoted cell death. Moreover, PUAO film displayed synchronous beating with mature cardiomyocytes showing high expression of cardiac specific α-actinin, troponin-T, and connexin-43 proteins [12]. Additionally, cultured cardiomyocytes on PUAO film expressed the physiological intracellular calcium functionality similar to mature cardiomyocytes [12].

Shin et al. used directed SAM to selectively trap target carbon nanotubes (CNTs) as an effort to control the growth of supramolecular hydrogel fibers and improve functionality of bioengineered cardiac tissues [117]. Surfaces of CNTs stimulate the formation of hydrogelators in the vicinity of the fiber constructs, which results in increased fiber formation, changes in network morphology, and increased mechanical properties. Subsequently, this can improve electrophysiological performance of cardiac tissue in terms of increased beating rate and lower excitation threshold [117, 163]. Besides CNTs, metallic nanoparticles, with their size-dependent properties, have shown promise in overcoming many of the current limits of cardiac tissue engineering. Li et al. reported a nanocomposite composed of gold nanoparticles (AuNPs) and a collagen matrix, which improved tissue growth via localized strength, thus enhancing the assembly of intercalated discs by β1-integrin-mediated signals [151]. In addition, 3D structures based on rigid CNTs scaffolds have been used to improve CMs viability, proliferation, and maturation, but they require undesirable invasive surgeries for implantation [164]. On the platform of 3D gel-based matrix, an injectable reverse thermal gel (RTG) functionalized with CNTs (RTG-CNT) that switches their morphology from a solution at room temperature to a three-dimensional (3D) gel-based matrix shortly after reaching body temperature was developed [164]. This extends long-term CMs survival, promotes CMs alignment and proliferation, or improves CM physiological function. Recently, Mason et al. reported a highly-ordered 3D fibrous protein scaffold derived from a self-assembly processes [153]. This resulted from a balanced system of low-entropy processes in which a set of interactions between different chain residues formed amorphous aggregates, thus mimicking self-assembling protein systems in nature. As an alternative to self-assembly, electrospinning produces nanofibers and nanofibrous structures from a broad range of biomaterials-based dopes in which advantages, drawbacks and potential applications are discussed in next sections.

### Electrospinning for 3D scaffold fabrication

**Fig. 3:**
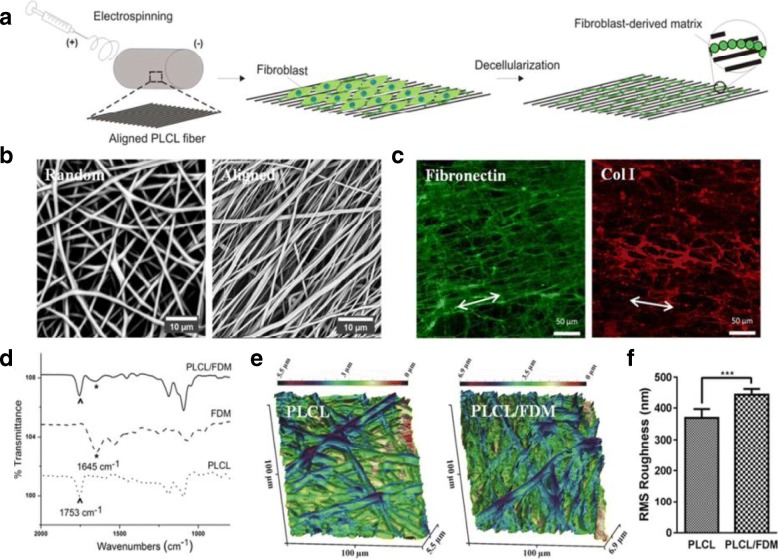
Fabrication and characterization of PLCL/FDM. **a** Illustration represents the fabrication process of PLCL/FDM. **b** Random and aligned orientations of PLCL fibers. Scale bar of SEM images is 10 μm. **c** Fibrillary ECM components in FDM were stained against FN and collagen type I. The direction of PLCL fiber alignment is shown by double headed arrows. Scale bar is 50 μm. **d** ATR-FTIR spectra of FDM with C=O at 1753 cm^− 1^ from PLCL and amide group at 1645 cm^− 1^ from FDM. **e** AFM images for surface topographical features of PLCL and PLCL/FDM; color scale shows their surface roughness and difference in height. **f** Quantitative comparison of root mean square (RMS) roughness calculated from AFM images. Statistical significance (***p < 0.001). The reproduced image is permitted from [45]

Electrospinning could be used to make nanofibers from a variety of polymers and it is well suited to 3D nano-scaffold constructs in cardiac tissue engineering [165]. In essence, the electrospinning technique is based on an electric field to create a charge on the surface of polymer solutions, thus generating a force opposing its surface tension and allowing fibers to be drawn out [166]. Many parameters can be used to tune this process, including electrical charges from the jet, solvent characteristics, length of polymers, flow rates, voltage levels, and collector distance; all of these considerations, and others, need to be taken into account to get a final polymer fiber in nanofibrous architecture [167, 168]. The resulting products are collected on solid or liquid substrates, or even substrate free, to form 3D micro-fibrous and nanofibrous scaffolds. Suhaeri et al. reported a new platform based on a fibroblast-derived, matrix-coupled, aligned and electro-spun nanofiber [45]. In their work, a hybrid scaffold structure composed of poly(l-lactide-co-caprolactone) (PLLA-PCL) and fibroblast-derived ECM (PLLA-PCL/FDM) was aligned to form an artificial cardiac microenvironment. The physical mechanical property of PLLA-PCL in the parallel direction shows the anisotropic nature of the aligned PLLA-PCL fibers. The PLLA-PCL/FDM was produced from the fibroblast culture on the PLLA-PCL fiber for 5–7 days and the ECM was collected from a subsequent decellularization. On this co-culture system, cellular characteristics of differentiation, phenotyping, cell viability, and maturation of H9c2 and neonatal rat CMs were significantly improved compared to those in fibronectin (FN)-coated electro-spun PLLA-PCL fibers (Fig. 3) [45]. On the aligned scaffold, cells spread along the directional cues instead of the random growth in every direction observed in the random scaffold. In addition, non-sulfated polysaccharides [169], biopolymers [170], and both organic and inorganic frameworks [171] have been integrated into PLGA to improve its biocompatibility and mechanical properties; and this highly depends on polymer concentration. However, due to collector plate constructs, nanofibrous scaffolds made from electrospinning are generally 2D; limiting their clinical relevance. Recently, a rotating cylinder has been demonstrated as a replacement for the collector plate used in electrospinning, which was utilized to produce a tubular scaffold and allow for growth factors to be released in a controllable fashion [172, 173]. A scaffold platform with polycaprolactone (PCL) nanofibers and vascular endothelial growth factor (VEGF)-encapsulated gelatin particles was fabricated to extend half-life time and stimulations of VEGF to mesenchymal stem cells (MSCs) and ECs [174]. In addition, paracrine mechanisms that are involved in MSC differentiation into cardiomyocytes are only limited to cell differentiation rates, not directly impacting to cell differentiation [175, 176]. Jiang et al. reported that this construct can drive the differentiation of MSCs to ECs and keep the stability of the tubular structure [174], indicating that growth factor (GF)-releasing scaffolds are potential platforms based on the electrospinning process for cardiac tissue engineering.

Recently, it has been shown that use of a Teas chart could provide useful information in terms of solubility and spin-ability for the electrospinning process [177–179]. Polymers should have solubility in the target condition, as values outside of a specific range will result in electro-sprayed beads and aggregates [177]. Higher fidelity nanoscale topography and bio-activity integration in the 3D architecture on the ECM-inspired nanofibrous scaffolds showed outstanding advantages for engineering 3D anisotropic cardiac tissues [137, 180].

### Thermally-induced phase separation

Thermally induced phase separation (TIPS) is another robust method to make 3D scaffolds. It involves five steps: polymer preparation, phase separation and gelation, solvent extraction, freezing, and freeze drying [181]. Once a polymer is dissolved in a specific solvent, the solution becomes thermodynamically unstable and results in two material phases: one “rich” in polymer and another phase “lean” in polymer. The resultant polymer structure depends on the ratio of polymer to solvent and conditions of the phase separation. Once the solvent is extracted, the phase of lean polymer is removed, and the polymer rich phase is identified as being in one of three categories: powder, closed cell foam, and open cell foam. Open cell foam is the type used to make 3D scaffolds for human chondrocyte growth and ECM formation [182]. ECM-derived porous foams are biologically-relevant substrates in advanced 3D in vitro cell culture models through controlling freezing and lyophilization procedures [183].

Luca et al. reported the formation of surface structures of TIPS-based scaffolds formed in water at room temperature [184]. The TIPS method allows for tuning of surface morphology which benefit tissue regeneration of preosteoblasts [184]. Peña et al. presented an injectable and biomimetic RTG that was functionalized with poly-L-lysine or laminin to promote longevity of cultured CMs, neonatal rat ventricular myocytes (NRVM), and adult rat ventricular myocytes (ARVM) [130]. Their results showed that the RTG functionalized with lysine stimulated NRVM grow and differentiated heart-like functional syncytia. Beating cells were recorded after 21 days in both cases of RTG and Lysin-functionalized RTG [130]. In addition, TIPS can be combined with porogen leaching to increase levels of architectural control. Porogen leaching (paraffin, sugar) can promote the formation of micropores with morphologies such as spherical, tubular, and disk shaped pores within the scaffold [185]. These micropores play important roles in enhanced cell penetration, proliferation, mass transport of nutrients, and growth factors in studies of angiogenesis and tissue formation. Several research groups have developed anatomically shaped molds with reverse solid freeform fabrication (SFF) in a PLLA solution [186, 187]. Architectural features were formed through three steps: ECM-mimicking materials, formation of pores for cell penetration and mass transport, and anatomical scaffold shaping. This last step is vital for structural tissue like bone and cartilage. TIPS can be used in concert with porogen leaching and 3D molds and with common chemical and biological polymers to create structural tissue scaffolds with excellent processing flexibility.

### Bioprinting for 3D scaffolds

Advancements in 3D printing have now begun to see its use in tissue engineering. State-of-the-art techniques in this field includes laser direct writing and multiphoton polymerization, which can be used for computer-aided scaffold design [188]. The process of designing and manufacturing scaffolds in this way includes several steps: design of functionally graded scaffolds, modeling of selective laser sintering and fused deposition modeling (FDM) processes, development of bioreactors, and 3D bioprinting [188–190]. Laser systems such as femtosecond- and ultraviolet-based sources allow for precise manufacture of 3D tissue scaffolds, which are engineered entirely through computer-aided design [191]. Zheng et al. reported the process of using computer-controlled UV laser systems for 3D scaffolds with many kinds of polymers such as polyethylene glycol diacrylate (PEG-DA), ormocomp, pentaerythritol tetra-acrylate (PETRA) [192]. More recently, a class of micro-architected materials with high-ordered structural connectivity and nanoscale features was printed by projection micro-stereolithography [192]. By using biopolymers, the technique could be used to produce biocompatible micro-lattices for soft tissue engineering, which are used as injectable scaffolds that can either induce endogenous cardiomyocyte repairing [193].

Seeded cardiomyocytes can be grown in hexagonal 3D fiber scaffolds made by melt electro-writing, a form of 3D printing. The resultant hexagonal microstructures have outstanding mechanical characteristics, allowing for large anisotropic reversible deformations; this deformable structure mimics microstructure of myocardial tissue [137]. Moreover, the high porosity of these structures aids formation of aligned tissues and are effective as cardiac patches on contracting hearts. These functional human myocardial patches feature properties highly desirable for clinically relevant cardiac repair [96]. As a result, iPSC-derived CMs have been successfully cultured in multi-cellular 3D bioprinting substrates for vascularized heart tissue [98]. Human umbilical vein endothelial cells (HUVECs) and iPSC-CMs have been encapsulated within hydrogel strands, containing alginate and PEG-fibrinogen, and forced out through custom microfluidic printing heads to form spatial depositions with high fidelity and resolution. Maiullari and colleagues have reported a 3D cardiac tissue composed of iPSC-CMs from different tailored geometries with a high orientation index [98]. Blood vessel-like shapes differentiated from HUVECs can be used for in vivo grafting, which is a better integrated support for engineered cardiac tissue [98]. These findings also bring important contributions to functional heart tissue generation in vitro through 3D PEG-fibrinogen hydrogels to recover their pluripotency [98]. This technique plays a key role in the design of printed micro-fibrous constructs used to assemble complex vascular networks. For example, bio-printed ECs following this can effectively develop vasculature in the transplanted tissues in the same manner of native vessels [194]. The results of bio-printed 3D vessel-based therapy directed to restore blood flow can counteract cell death and promote regeneration in the revascularization of ischemic or damaged organs, which highly relies on microenvironment engineering for supplies of oxygen and nutrient.

However, due to the lack of oxygen and nutrient diffusion (in the 100–200 μm scale) in porous structures, migration of iPSCs tends to be in the outer zone of hydrogels; and this produces inhomogeneous cellular distribution in vascular networks in vivo [195, 196]. These diffusion problems could be solved via an integrated system of porous structures and parallel fibers to form an engineered vascular network. By addition of 1% w/w PEG-DA monomer to bioprinting materials, the homogeneous culture biosystem fully supplies nutrients to all regions of the 3D constructs [98] . This technique has been used for iPSC-derived CMs culture to produce myocardial-like tissue [98] and generate 3D vascular structure [197]. Alternatively, circulation in the 3D constructs is supplied by a microfluidic device bearing a Y-junction (2 inlets, 1 outlet) in which the flows of two different bio-inks are precisely driven by external microfluidic pump [98]. Interestingly, this construct showed great promise for artificial skeletal muscle generation once the dimensions of channel were reduced to 500 × 500 μm^2^ (cross-section) to create an extremely-small dead volume (< 2 μL); this allowed rapid tuning between the two bio-inks during printing. This system also allows building heterogeneous structures composing of iPSC-derived CM and HUVEC could potentially mimic native cardiac contraction in better than those described above.

Functional contraction of the myocardium is orchestrated by electrical stimulation propagation in the right sequence and is driven partially by CM spatial orientation; therefore, proper orientation is a critical goal for organization of CMs [98, 159]. The organization of CMs embedded in 3D bio-printed fiber structures is impacted by the surrounding fiber matrix direction; often, growth of iPSC-derived CMs is directed along the fiber printing direction. Contraction can be further enhanced with higher material conductivities. Scaffolds that couple electrical and elastic materials have become valuable for cardiac cell function, but current conductive materials do not show tunable physiological properties for cell behaviors [138, 198]. Electrospun conductive scaffolds were reported of use in cardiac tissue engineering for enhancement of connexin 43 expression [96, 198]. By integration of AuNPs into hydrogel scaffolds, the polymer templated gel becomes tunable with a Young’s modulus similar to that of myocardium, polyaniline, and polypyrrole. Neonatal rat CMs were cultured on the scaffold and expressed high level of connexin 43, with or without electrical stimulation. Hosoyama et al. have also reported a novel nanoengineered hybrid electro-conductive cardiac patch for treating the infarcted myocardium [96] of which classification and localization from medical images are detected by machine learning [199–203].

## Machine learning and precision control for 3D scaffold fabrication

### Machine learning in tissue platform

As mentioned, currently the most obvious use of machine learning (ML) in this field is identifying patterns in tissue-related data and/or classifying specific tissue constructs. One example of a problem of interest is that of classifying the phenotype of differentiated, stem cell-derived CMs. One group sought to classify CM phenotype by matching distinct groups of shapes with distinct groups of action potential waveforms [204]. It was done by staining the cells of interest, optically mapping them during contraction, converting time-varying pixel intensity to discrete waveforms, and then using ML algorithms to identify groupings of AP behavior which they could compare to cell cluster shape data. The employed ML is what’s known as spectral clustering whose algorithm attempts to minimize a “similarity” weight value between sets of inputs, thereby grouping them [205]. In this case, the authors used aligned and averaged AP as the input to the clustering algorithm, allowing the algorithm to minimize similarities between groups of the AP waveforms, and then mapped these groupings to cell cluster spatial distributions. These methods have been successfully applied in biomedicine and cell biology with various stage-of-the-art machine-learning algorithms [58, 60, 206].

**Fig. 4:**
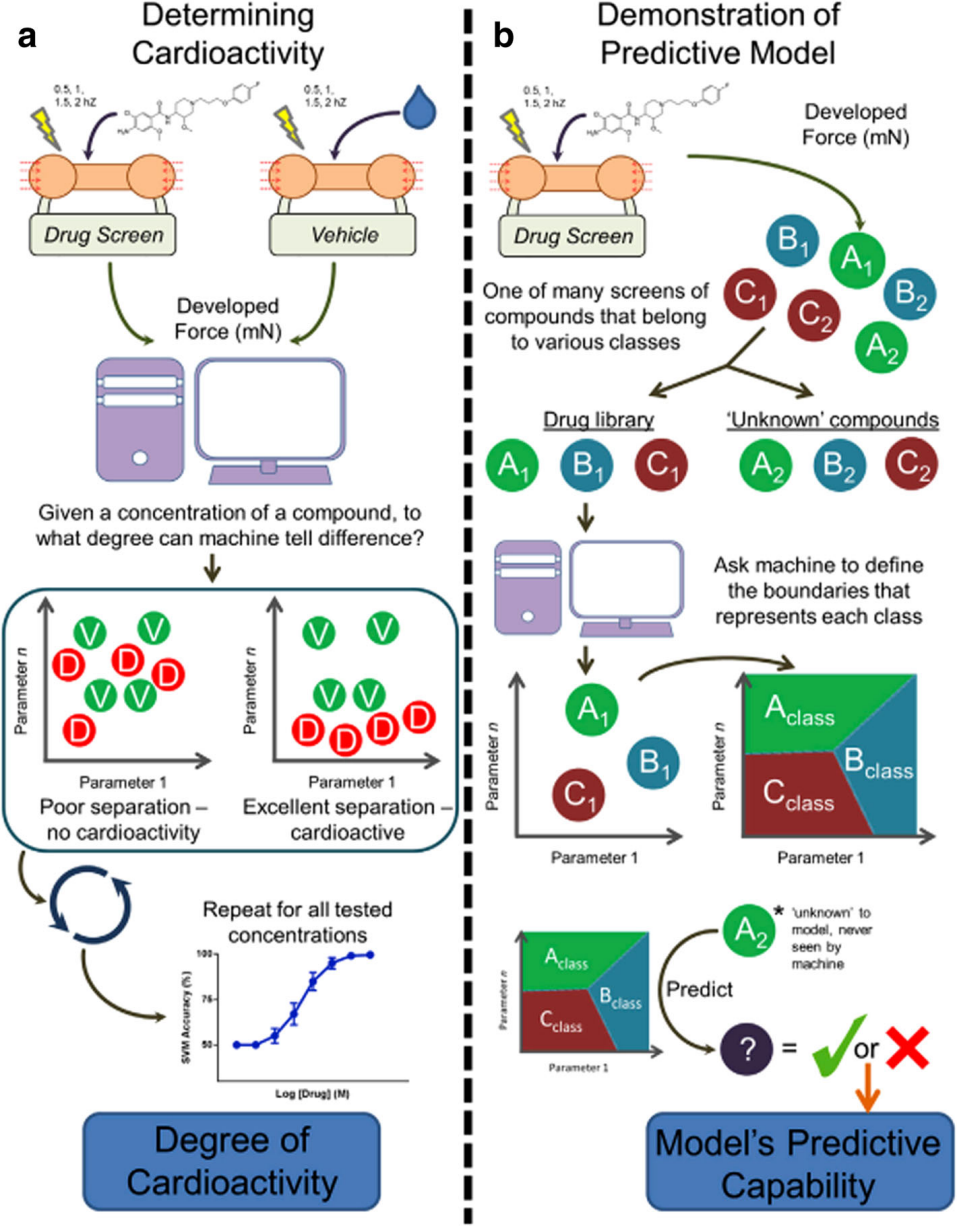
Machine learning for drug screening on human iPSCs-derived engineered cardiac tissue. **a** Waveform pattern parameters are determined based on concentration of cardioactive compounds compared to the binary support vector machine (SVM). The collected data points would be in line with those of vehicle as if the compound does not modulate the contractile behavior of human ventricular cardiac tissue strips (hvCTSs). If data of cardio active effects are more distinguishable, it shows in a higher SVM accuracy which is possible to separate two compound groups. The degree of cardio activity of a given concentration for target compound is shown in a singular quantitative index with the binary SVM approach. **b** Library of compounds is built on a model for prediction of mechanistic action of screened compounds. Data from the library group allow the machine learning defines boundaries of various drug families. Finally, the developed model can be applied for the unknown compounds on tissue engineering. The image is reproduced with permission from [41]

A more-recent example of ML used in this space was geared toward not only classification of cardiac tissue contractile events [207] but extending this classification set into a predictive model for preclinical screening effects of drugs on cardiomyocyte function [41]. The predictive models are highly dependent on machine learning methods such as naïve Bayesian, support vector machines (SVM), and end-to-end (E2E)-integrated ML system [53], of which they are leveraged by bigger datasets generated from high-throughput screening data. Lee et al. reported a SVM to develop a drug screening assay on hiPSCs-derived cardiac tissue **(**Fig. 4) [41]. In this approach, groups of linearly separable data were demarcated by planes in order to classify them [208]; and the planes themselves were statistical maximizations of group separation based on feature points (i.e. support vectors), rather than the more-computationally intensive nearest-neighbor piecewise approach [209].

They first qualified models by generating force data and derived parameters from stimulated cardiac cells, mixing the data with a control set, allowing a binary SVM to attempt to classify the data, and then quantifying the resulting SVM accuracy [210]. This classification model accuracy then becomes a proxy for cardiac activity of the drug. About 50% accuracy means that the SVM could not separate control from drug but accuracy greater than 50% indicates that the statistical model was able to group the drug and control outputs into different regions of the parameter space and, therefore, declare a difference in behavior [41, 211]. Data of cardio active effects express in a higher SVM accuracy, if they are more distinguishable from two compound groups. Based on a given concentration, the degree of cardio activity for a target compound is shown in a singular quantitative index with the binary SVM approach [41, 207]. Next, a library of this drug screen testing data was combined and an SVM designed for multiple classes was used to define parameter space regions for each. The library of compounds was built on a multiple-category prediction model for mechanistic action of screened compounds and chemogenomic databases [212, 213]. Data from the library group allow the machine learning defines boundaries of various drug families and mechanism of action [214]. Finally, the developed model can be applied for the unknown compounds on tissue engineering. After doing so, a withheld data set of the same form was fed into their predictive model to see if the SVM could properly classify drug interactions [215], integrating multiple omics data [216], and unknown drug compounds [217]. In their demonstration, they were able to classify cardiac activity of unknown compounds with an accuracy of roughly 72% and generalize the results to other drug families with an accuracy above 70% [218]. Further, ML and its myriad algorithms can also be used on the protein and gene side of tissue engineering, as it has been demonstrated or proposed for histopathological image analysis [43], ligand affinity [42], folding structure [219], gene expression and biomarker data mining [220, 221], and in evaluation of pre-implantation embryos [222]. Large datasets such as the “Tissue Atlas” [223], a human proteome map categorized by tissue, could easily be used as a training and testing set for ML algorithms targeting identification of impaired tissue or disease onset.

### Precision control in fabrication of 3D scaffold

The ever-widening and accelerating field of robotics both contributes to and has the possibility of benefitting from tissue engineering. The contribution of robotics to tissues engineering lies mostly in the manufacturing space; as automated fabrication has hastened tissue construct research. Of particular popularity at the moment is the concept of robotic bio-fabrication, also known as organ printing or bioprinting. Bioprinting was defined by members of the first international workshop on the subject in 2004 as the “*use of material transfer processes for patterning and assembling biologically relevant materials—molecules, cells, tissues, and biodegradable bio-materials—with a prescribed organization to accomplish one or more biological functions*” [224]. In other words, it’s the use of automated fabrication to faster transfer from the scaffold design and tissue culture, to clinical settings, especially in the field around regenerative cardiomyocytes.

As discussed earlier, 2D and 3D cardiomyocyte cultures in biomimetic conditions are crucial to the improvement of knowledge surrounding cardiac tissue development [225]. Researchers have presented methods for forming these tissue constructs in a variety of manners— from using electrospinning to create scaffolds enabling cell attachment and growth [96] to 3D patterning of tissue-similar constructs [226], or using printer deposited spheroids to induce scaffold-less self-assembly of tissue [227, 228], although some of these technologies have significant hurdles to overcome still. Within the last decade, researchers have begun to concern themselves with the systems design of holistic industrial bio-fabrication lines, including the design stage prior to and maturation stage after bio-fabrication [229]. In-vivo bio-fabrication is also getting attention; beyond bioresorbable printed scaffolds [230], there have even been demonstrations in mice of laser printing of photoactive resins above the calvaria to form bone-like caps [230], which was integrated with the robotic controlling.

Tissue engineering is also feeding back into robotics in two important ways—inspiring bio-mimetic robotic systems [231] and becoming a potential component within robots themselves [232]. Most bio-similar robots up to this point have focused on the use of soft materials to grip and move, as the field has acknowledged that the limited conformability of robotics prior to this trend is directly counter to the variety of conformable structures seen in nature [231]. Much of the interest in artificial tissue has been focused on muscle. One group demonstrated artificial muscle composed of polymer-based composites which bend and flex under cation exchange [233], similar to action potential propagation in cardiac tissue. Another group demonstrated this same concept using a collagen gel filled with rat CMs and initiated contractile behavior strictly chemically, using epinephrine and nifedipine [234]. This is somewhere between the former and latter contributions of tissue engineering but there are recent examples in which robotics systems have been designed from the systems level to take advantages of engineered tissues, themselves being bio-similar robotic systems. As an example of engineered tissue integrated robotics, researchers have demonstrated actuators which are comprised of myoblast-filled hydrogels and triggered by electrical stimulation [235], antagonistically contracting against each other to create both contraction and extension. It is of note here that not only are the actuators themselves engineered tissues, but they have been attached to their skeletal frame by culturing methods, and even the mechanical systems design mimics natural tissue. It is likely that more bio-similar, bio-integrated robotic hybrids are on the horizon.

## Conclusions

Cardiac tissue engineering has benefited greatly from advances in genetic engineering, material engineering, electrical engineering, and biochip design. Within genetic engineering, genome editing is a pioneering tool that has been used in the generation of new cellular, tissue and animal models to investigate cell-cell adhesion, differentiation of hiPSCs, and generation of CMs for various cardiac disease. However, the post-mitotic nature of CMs and various technical barriers present hurdles for bringing engineered cardiac tissue directly to therapeutic applications. Other cells such as cardiac fibroblasts, ECs, and muscle cells can potentially substitute for CMs in developing tissues for cardiovascular diseases.

One major technical advancement in this field is the ability to design a physical framework of biocompatible materials and the control of mechanical characteristics, which can be applied clinically. Due to the nature of CMs, scaffolds used for CM growth should be readily tunable for alignment/organization to produce efficient contractions. Further, electrical stimulation should be integrated into the system to perform intensity training in the later stages of CM culture [111]. This enables the connection of native and differentiated cells, at single cell levels of cellular communications, between hiPSC and CMs. Communication between CMs and their micro-environment within the engineered tissue should be understood in tandem with development of 3D biomimetic scaffolds and bioreactors in order to promote cost-effective scale-up of tissue production.

There exists a variety of supporting technologies which could be applied in the process of tissue engineering. One possibility is that machine learning be used involved in the design and processing of micro-physiological systems. High-throughput fabrication could be optimized via scaffold geometry, cellular paracrine factors, and cellular communication, in order to maximize survival rates and completely functionalize engineered cardiac tissue. At the molecular and cellular level, engineered cardiac tissue derived from the HLA-null line should be tailored towards developing immune-resistant modified hiPSC-derived CM lines; this can be done using genome editing tools focused on solving cryopreservation general implantation issues.

Confucius said, “*Our greatest glory is not in never failing, but in rising every time we fail.”* We believe with focused and continued progress achieved by scientists across a range of multidisciplinary fields, cardiac tissue engineering will soon be viable for clinical use.

## Data Availability

N/A.

## Abbreviations

124 polymer: Poly(octamethylene maleate (anhydride) 1,2,4-butanetricarboxylate)
AHM: Artificial heart muscle
ARVMs: Adult rat ventricular myocytes
AuNPs: Gold nanoparticles
Cas9: Caspase 9
CM: Cardiomyocyte
CNTs: Carbon nanotubes
CPVT1: Catecholaminergic polymorphic ventricular tachycardia type 1
CRISPR: Clustered regularly interspaced short palindromic repeats systems
CSCs: Cardiac stem cells
ECM: Extracellular matrix
ECs: Endothelial cells
FDM: Fused deposition modeling
FN: Fibronectin
GF: Growth factor
HDR: Homology directed repair
hiPSC-CMs: Human induced pluripotent stem cells-derived CMs
hiPSCs: Human iPSCsHuman iPSCs
hMSCs: Human mesenchymal stem cells
HUVECs: Human umbilical vein endothelial cells
IL-1: Interleukin 1
IL-1Ra: Interleukin 1 receptor antagonist
iPSC-CMs: iPSCs-derived cardiomyocytes
iPSCs: Induced pluripotent stem cells
ML: Machine learning
MNPs: Magnetic nanoparticles
MSCs: Mesenchymal stem cells
NHEJ: Non-homologous end joining
NRVMs: Neonatal rat ventricular myocytes
PAM: Protospacer adjacent motif
PCL: Polycaprolactone
PEG-DA: Polyethylene glycol diacrylate
PETRA: Pentaerythritol tetra-acrylate
PGA: Polyglycolic acid
PLA: Poly(L)-lactic acid
PLLA: Poly(L-lactide)
PUAO: Elastomeric antioxidant polyurethane
ROS: Reactive oxygen species
RTG: Reverse thermal gel
RYR2: Ryanodine receptor 2
SAM: Self-assembled monolayers
SFF: Solid freeform fabrication
sgRNA: Single-stranded guide RNA
TIPS: Thermally induced phase separation
VEGF: Vascular endothelial growth factor
YAP: Mechanosensitive transcriptional activator
